# Controlling Cancer Cell Behavior by Improving the Stiffness of Gastric Tissue-Decellularized ECM Bioink With Cellulose Nanoparticles

**DOI:** 10.3389/fbioe.2021.605819

**Published:** 2021-03-17

**Authors:** Jisoo Kim, Jinah Jang, Dong-Woo Cho

**Affiliations:** ^1^School of Interdisciplinary Bioscience and Bioengineering, Pohang University of Science and Technology, Pohang, South Korea; ^2^Department of Mechanical Engineering, Pohang University of Science and Technology, Pohang, South Korea; ^3^Department of Convergence IT Engineering, Pohang University of Science and Technology, Pohang, South Korea; ^4^Institute of Convergence Science, Yonsei University, Seoul, South Korea

**Keywords:** 3D cell-printing, cellulose nanoparticles, tissue engineering, gastric-derived extracelluar matrix bioink, 3D gastric cancer model

## Abstract

A physiologically relevant tumor microenvironment is favorable for the progression and growth of gastric cancer cells. To simulate the tumor-specific conditions of *in vivo* environments, several biomaterials engineering studies have investigated three-dimensional (3D) cultures. However, the implementation of such cultures remains limited because of challenges in outlining the biochemical and biophysical characteristics of the gastric cancer microenvironment. In this study, we developed a 3D cell printing-based gastric cancer model, using a combination of gastric tissue-specific bioinks and cellulose nanoparticles (CN) to provide adequate stiffness to gastric cancer cells. To create a 3D gastric tissue-specific microenvironment, we developed a decellularization process for a gastric tissue-derived decellularized extracellular matrix (g-dECM) bioink, and investigated the effect of the g-dECM bioink on promoting the aggressiveness of gastric cancer cells using histological and genetic validation methods. We found that incorporating CN in the matrix improves its mechanical properties, which supports the progression of gastric cancer. These mechanical properties are distinguishing characteristics that can facilitate the development of an *in vitro* gastric cancer model. Further, the CN-supplemented g-dECM bioink was used to print a variety of free-standing 3D shapes, including gastric rugae. These results indicate that the proposed model can be used to develop a physiologically relevant gastric cancer system that can be used in future preclinical trials.

## Introduction

Gastric cancer is the fourth most common cancer, and the second most common cause of cancer-related death worldwide (Spolverato et al., 2015). In Western countries, more than 80% of patients that are diagnosed with advanced gastric cancer have poor prognosis. As a result, the 5-year survival rate for this disease is under 30% (Roukos, 2000). To date, surgical therapy is the only approach that completely eliminates local tumors; however, the opportunity to remove a patient’s tumor is often lost, as diagnosis occurs too late (Spolverato et al., 2015). Patients with advanced stage gastric cancer receive chemotherapy as well as adjuvant or neoadjuvant therapy. Although this approach achieves improved therapeutic effects, survival rates remain unsatisfactory because of the tumors’ high drug resistance (Yuan et al., 2017).

As the progression and growth of gastric cancer is influenced by the tumor microenvironment (da Cunha et al., 2016; Jang et al., 2018), establishing a physiologically relevant microenvironment is increasingly important in *in vitro* study. In particular, the extracellular matrix (ECM) surrounding cancerous growths regulates cellular functions such as migration and proliferation, through both cell–cell and cell–ECM interactions, which further affects cancer progression and aggressiveness (Crotti et al., 2017). Moreover, a decellularized tissue ECM (dECM) provides a tissue-specific microenvironment for the cells, and directs cellular behavior in cancerous growths (Hoshiba, 2019; Ferreira et al., 2020). Although several naturally-derived biomaterials such as collagen and Matrigel have been used for mimicking the cancer system (Jang et al., 2017), these purified materials find it difficult to recreate the substrata of their intrinsic environment (Tian et al., 2018). In this respect, development of biomaterials can provide cancer-specific microenvironmental components and compositions, which are essential in regulating *in vivo*-like cellular behaviors.

Recently, several studies have demonstrated that decellularized extracellular matrixes promote cancer cell behavior (Rijal and Li, 2017; Jin et al., 2019); a lung-derived decellularized ECM enabled the demonstration of cancer cell proliferation, with its morphological differences inducing the aggregation of cancer cells (Tian et al., 2018). Furthermore, through its control of the integrin-mediated pathway, ECM stiffness has a high potential to regulate the activation of cancer cell signaling (Seewaldt, 2014); with an increase in matrix stiffness, the promotion of integrin β1 clustering and the activation of β-catenin were observed, leading to an escalation of invasion and metastasis behaviors. Diverse attempts have been made to achieve sufficient mechanical strength for bioengineered matrixes, including increasing hydrogel concentration, or reinforcing the material by adding cellulose nanoparticles (CN), which are the most widespread natural material that have biocompatible characteristics (Jang et al., 2018; Athukoralalage et al., 2019). However, these approaches are yet to be studied in detail for the development of biochemically and biophysically related materials. Tissue-specific biomaterials and the regulation of matrix stiffness are crucial, as they can enable a more comprehensive assessment of gastric cancer cell responses by simulating the real microenvironment.

In this study, we introduce a mechanically reinforced bioink, consisting of gastric dECM (g-dECM) and CN, that models a biochemical microenvironment characteristic of gastric cancer. Moreover, CN enables the modulation of matrix stiffness, thereby achieving improved biophysical features. In addition, using a three-dimensional (3D) cell printing system, we fabricated 3D structures, including a mimic of a gastric ruga, using cell-laden bioink. Finally, we observed enhanced cancer-related characteristics such as cell aggregates, cellular interactions, and drug resistance in the developed bioink, compared with Matrigel and collagen.

## Materials and Methods

### Decellularization of Gastric Tissue

Fresh porcine gastric tissue was obtained from a butcher shop (Pignara). Before starting the decellularization process, the mucosa layer was removed from the porcine gastric gland, cut into approximately 0.5-mm-thick slices, and washed with distilled water for 1 h to remove any remaining blood. The sliced tissues were then rinsed in a 25 mM 1 wt% sodium dodecyl sulfate (Thermo Fisher Scientific) solution for 24 h, and 25 mM 1% Triton X-100 solution (Sigma-Aldrich) for another 24 h, to remove residual cells. The tissues were subsequently treated in PBS for 24 h to wash the chemical detergents, and sterilized in 0.1 w/v% peracetic acid solution for 1 h. Following this, they were washed with PBS and distilled water for 30 min. Thereafter, decellularized gastric tissues were deep frozen at −80°C and lyophilized for 48 h. A g-dECM pre-gel solution was prepared by digesting 200 mg of the ground g-dECM powder in a solution of 0.5 M acetic acid (DUKSAN) supplemented with 20 mg of pepsin (Sigma-Aldrich), and stirring vigorously for 72 h. The biochemical characteristics of the g-dECM were evaluated using the remaining DNA, collagen, and glycosaminoglycan (GAGs), as described previously (Pati et al., 2014).

Before using the g-dECM bioink in experiments, the pH was adjusted to 7.4 by adding a 10 M NaOH solution, for thermal gelation. The g-dECM bioink and NaOH solution was preserved in ice during this pH adjustment process, to prevent gelation before use.

### Preparation of Cellulose Nanoparticles

The aqueous suspensions of CN were prepared using a modified protocol from the literature (Kumar et al., 2017). In brief, acid hydrolysis was performed by stirring microcrystalline cellulose (MCC, Sigma-Aldrich) with a 64 wt% H_2_SO_4_ solution at 45°C for 60 min. This reaction was quenched with the addition of cold distilled water. The chilled solution was centrifuged several times, and dialyzed in distilled water, using snake skin (Thermo Fisher Scientific), to remove the acidic solution. The prepared aqueous suspensions of CN were stored at 4°C for further use.

### Transmission Electron Microscopy

The morphology of the CN was examined by transmission electron microscopy (TEM, JEM-1011, Jeol). The aqueous suspensions of CN were diluted to 0.1 wt% and dropped onto the surface of a thin carbon film-coated copper grid. The sample was dried overnight, following which, TEM analysis was performed at an accelerating voltage of 100–120 kV.

### Preparation of CN-Supplemented g-dECM Bioink

To prepare the CN-supplemented g-dECM bioink (CN-g-dECM bioink), CN solution was added to the 2% g-dECM bioink in a 1:40 ratio. The final concentration of the CN-supplemented bioink was varied in the range 0.01–0.5 wt% by adjusting the dilution of the aqueous CN solution prior to combination with g-dECM. This combination was mixed by applying over 70 cycles of gentle pipetting, to ensure the distribution of CN in g-dECM was uniform. In addition, to characterize the effect of CN on cellular behavior, we also created a g-dECM bioink control without CN (0% CN-g-dECM) for use in experiments.

### Rheological Characterization

The rheological properties of the g-dECM bioink were characterized using a rheometer (DHR-2, TA Instruments) with a 20 mm-diameter plate. To determine its viscosity, a steady shear sweep analysis of the pre-gel bioink was performed at 15°C. Dynamic frequency sweep examinations were performed to analyze the material’s frequency-dependent storage (G) and loss (G″) moduli at a 2% strain in the range 0.1–100 rad s^–1^ after incubation for 30 min at 37°C.

Rheological assessment of the CN-g-dECM bioink was performed similarly; dynamic frequency sweeps were conducted to measure the material’s frequency-dependent storage (G) and loss (G″) moduli at a 2% strain in the range 0.1–100 rad s^–1^ after incubation for 30 min at 37°C, and treatment with 100 × 10^–3^ M calcium chloride (CaCl_2_) solution.

### 2D/3D Cell Culture

Gastric cancer cell lines (AGS, SNU-1, and KATO3, Korean Cell Line Bank, South Korea) were cultured in RPMI 1640 (Gibco) supplemented with 10% FBS (Gibco) and 1% penicillin/streptomycin (Gibco). For the 3D cell culture, each cell line was encapsulated in g-dECM bioink, CN-g-dECM bioink, collagen, and Matrigel (Corning). The cell-printed g-dECM bioink, collagen, and Matrigel were fabricated and gelated by incubating at 37°C for 30 min. The cell-printed CN-g-dECM was crosslinked via treatment with 100 × 10^–3^ M CaCl_2_ solution and then incubated with the printed structure at 37°C for 30 min. Every cell-laden hydrogel was refreshed with a cell culture medium every other day and harvested for further analysis.

### Cell Viability Assay

Cell viability was evaluated by staining with Calcein AM and ethidium homodimer-1 solution (LIVE/DEAD Viability/cytotoxicity Kit, Thermo Fisher Scientific) following the instructions provided by the manufacturer.

### Quantitative Polymerase Chain Reaction (qPCR)

The total RNA from collected hydrogels was isolated using the GeneJET RNA Purification Kit (Thermo Fisher Scientific) following the manufacturer’s instructions. Complementary DNA (cDNA) was synthesized using the Maxima First Strand cDNA Synthesis Kit (Thermo Fisher Scientific) according to the manufacturer’s instructions. Gene expressions were then analyzed with SYBR Green PCR Master Mix and StepOnePlus real-time PCR system (Applied Biosystems). The fold changes of the target genes were calculated using the 2^–ΔΔCt^ method by normalizing them with the housekeeping gene (GAPDH) expression. Coding sequences for GAPDH, matrix metalloproteinase-2 (MMP2), catenin beta-1 (β-catenin), and integrin beta-1 (integrin β1) were designed using the National Center for Biotechnology Information reference sequences (Table 1) and Primer Express software v3.0.1 (Thermo Fisher Scientific) for preparing primers.

**TABLE 1:** Primer sequences for qRT-PCR.

Target gene	Primer
GAPDH	CTCCTGCACCACCAACTGCT
	GGGCCATCCACAGTCTTCTG
MMP2	CGTCTGTCCCAGGATGACATC
	ATGTCAGGAGAGGCCCCATA
β-catenin	GATACCCAGCGCCGTACGT
	GACCCCCTCCACAAATTGC
Integrin β1	CAACACCAGCTAAGCTCAGGAA
	CTAAATGGGCTGGTGCAGTTC

### Histological Analysis

To perform hematoxylin and eosin (H&E) staining, all cell-laden hydrogels were incubated in 10% buffered formalin solution for 30 min, washed three times with PBS, and fixed with paraffin. The hydrogels were subsequently sectioned to 30 μm slices using a Reichert-Jung 2035 microtome and placed on glass slides. The sections were immersed in xylene I and xylene II solution for 5 min each to remove the paraffin, then immersed in 100, 95, 80, and 75% ethanol solution, for 3 min each, and rinsed with distilled water for 5 min, for hydration. Then, sections were placed in hematoxylin solution for 10 min, washed with running tap water for 2 min, and placed in 1% acid alcohol solution for 5–30 s. To complete staining, the sections were immersed in eosin solution for 2 min, washed with running tap water for 1 min, and placed in ethanol solutions (70, 95, and 100%) for 2 min each, for dehydration. Finally, sections were immersed in xylene I and xylene II solution for 5 min each, and the glass slides were sealed with a coverslip, using Permount Mounting medium (Thermo Fisher Scientific). The H&E-stained samples were visualized with a microscope. The sizes of the cell aggregates in H&E images were measured using the analysis tools in ImageJ software version 1.47 (National Institute of Health, United States). The size of cell aggregates in an experimental group was subsequently calculated as the average size of the measurement from three different samples.

### Immunostaining

To perform immunofluorescence staining experiments, all cell-laden hydrogels were fixed with 10% buffered formalin solution for 30 min, washed three times with PBS, and permeabilized with 0.1% Triton X-100 in PBS for 15 min. Next, hydrogels were stained with Alexa Fluor^TM^ 594 phalloidin and 4,6-diamidino-2 phenylindole (DAPI) (Thermo Fisher Scientific) and examined with a laser confocal microscope (Leica TCS SP5 II).

### Drug Resistance Testing

Gastric cancer cell-laden hydrogels were cultured in fresh media for 2 weeks. The media were supplemented with 5-Fluorouracil (5-FU, Sigma-Aldrich) after 3 days of culturing. Cell viability was determined using WST-8 [2-(2-methoxy-4-nitrophenyl)-3-(4-nitrophenyl)-5-(2,4-disulfophenyl)-2H-tetrazolium, monosodium salt] cell proliferation assays from an assay kit (Cell Counting Kit-8, Dojindo Molecular Technologies). The treatment responses for each culture condition were normalized to that of non-treated cultures. In addition, the IC_50_ value was calculated using GraphPad Prism 7 software.

### 3D Cell Printing Using the Gastric Cancer Cell-Laden g-dECM Bioink

To print the *in vitro* gastric cancer structures, we used a previously developed extrusion-based 3D cell-printing system named the Integrated Composite tissue/organ Building System (ICBS) (Supplementary Figure S1; Kim et al., 2017). The bioinks were prepared by encapsulating gastric cancer cell lines (cell concentration: 5 × 10^6^ cells mL^–1^) with 2% g-dECM pre-gel solution into each hydrogel. A grid pattern, rectangular shape, and gastric ruga shape were manufactured using the in-house developed 3D cell printing system with the 2% g-dECM bioink. The printing was performed at 15°C using a 300 μm nozzle, and the speed of the pushing motion was regulated in the range 20–70 kPa using the Nano Master SMP-III (Musashi Engineering, Ltd.). All printed structures were incubated at 37°C for 30 min and refreshed with a cell culture medium every other day.

### Statistical Analysis

In this paper, statistical data is expressed as mean ± standard error. The Student’s *t-test* was conducted to compare two different experimental groups, whereas one-way analysis of variance was performed to compare more than two different experimental groups. These procedures were followed by *post hoc* analysis using Tukey’s multiple comparisons test. Values were considered significant at *^∗^p* < 0.05, ^∗∗^*p* < 0.01.

## Results

### Preparation and Characterization of the Gastric Decellularized Extracellular Matrix-Derived Bioink

**FIGURE 1:**
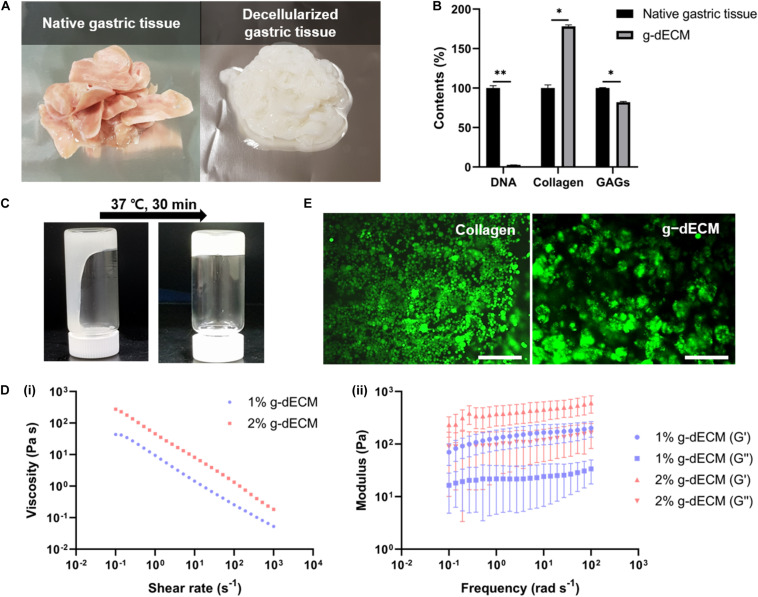
Preparation and characterization of g-dECM bioink. **(A)** Optical micrographs of sliced native gastric tissue and decellularized gastric tissue (g-dECM). **(B)** Comparison of the amount of extracellular components (collagen, GAGs) and DNA in native tissue and g-dECM. **(C)** Sol-gel transition of the g-dECM bioink. **(D)** Rheological properties of g-dECM bioink: (i) viscosity at 15°C (ii) dynamic modulus at 37°C. **(E)** Live/dead evaluation of human gastric cancer cell-line (AGS) on day 14 (scale, 200 μm, *p < 0.05, **p < 0.01).

We successfully developed processes for the preparation of g-dECM from native gastric tissue (Figures 1A,B). Our method for developing g-dECM removes cellular material from tissue while minimizing ECM loss and damage. This was validated through a DNA quantification assay, which determined that <37 ng/mg of dsDNA remained in the g-dECM, 2.7 ± 0.3% of the quantity in native tissue, whereas the collagen and GAG concentrations in the g-dECM were 173 ± 3% and 80 ± 3% of the contribution to the content of native tissue (Figure 1B). For effective decellularization, the quantity of cellular components should be less than 3% of the native tissue, and less than 50 ng/mg in the dECM (Pati et al., 2014). These results thus indicate that we effectively decellularized gastric tissue while preserving ECM components.

For cell culture, the pH of the g-dECM bioink is adjusted using NaOH (Figure 1C). When incubated at 37°C for 30 min, the pH-adjusted g-dECM bioink showed a hit-induced sol-to-gel transition in response to temperature changes. Before performing 3D cell culture, we measured the shear viscosity and storage/loss modulus of pH-adjusted g-dECM bioink to ensure its suitability for extrusion-based printing, and verify the shape retaining ability of printed structures. Both 1 and 2% g-dECM bioink showed a shear thinning behavior, wherein the viscosity of the bioink decreased as the shear rate increased (Figure 1Di). Such shear thinning behavior is vital for 3D cell-printing techniques, because it enables the dispersal of the bioink during printing. Further, after incubating at 37°C for 30 min, the storage modulus was higher than the loss modulus for both bioinks, indicating that they can retain their shape (Figure 1Dii), a critical factor for the fabrication of 3D cell-printed constructs (Pati et al., 2014). However, the 2% g-dECM demonstrated higher mechanical stability metrics than the 1% g-dECM bioink, suggesting that it is more suited to 3D cell culture.

To evaluate the toxicity of the developed g-dECM bioink—a fundamental aspect of developing biomaterials (Stoddart, 2011a,b)—we examined the cell viability of the g-dECM bioink with reference to that of collagen. In these experiments, we encapsulated 3D printed cell constructs using AGS, a gastric cancer cell line, in the pH-adjusted g-dECM bioink, and in collagen. Over 95% cell viability was observed with both groups on day 14 (Figure 1E), indicating that the g-dECM bioink is non-cytotoxic, given its similar response to the Type I collagen hydrogel.

### 3D Printing of Gastric Cancer Cells

**FIGURE 2:**
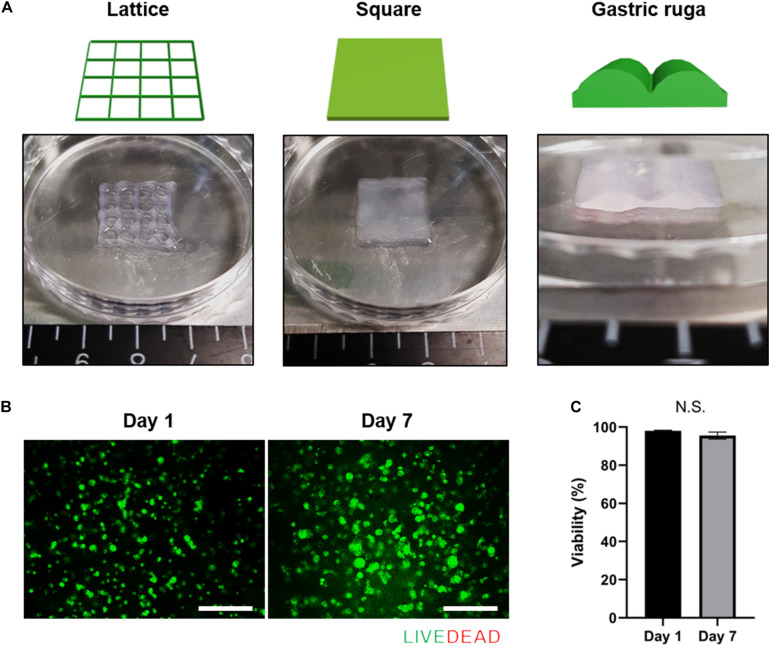
Application of g-dECM bioink to 3D cell printing of a variety of constructs. **(A)**. Heterogeneous structures 3D cell printed in accordance with modeling. **(B)** Live/dead evaluation of the gastric cancer construct 1 and 7 days after culturing. **(C)** Viability of cells in construct 1 and 7 days after culturing (scale, 200 μm; N.S., no significance).

3D cell printing is a promising tool for fabricating arbitrary shapes, and placing cells in designated locations simultaneously (Pati et al., 2014; Yi et al., 2019; Kim et al., 2020). To confirm its suitability for 3D cell printing, we measured the fidelity of shapes created using cell-laden g-dECM bioink (Figure 2A), demonstrating that it could print a pre-designed grid and rectangular patterns. Further, the gastric ruga pattern designed to mimic the shape of gastric tissue in the macro scale, was printed accurately. As it has been shown that the shear force during printing can damage the cells and reduce cell viability (Derby, 2012), we verified the cell viability after 3D cell printing. The viability of KATO-III in the printed structure was found to be sufficiently high (>95%) (Figures 2B,C) 1 and 7 days after cell printing, demonstrating that the developed bioink can be used not only for fabricating complex structures, but also for culturing various types of gastric cancer-related cells.

### Response of Cellular Behavior Based on the Microenvironment

An *in vitro* aggregated cancer model that mimics the more realistic *in vivo* conditions (Adenis et al., 2020) has been demonstrated. In addition, *in vitro* cancer models that support tissue-specific function and cell aggregation using a decellularized tissue ECM have also been reported (Rijal and Li, 2017; Tian et al., 2018). As the cellular function and drug resistance of cancers are related to cell aggregation (Jianmin et al., 2002; Zhang et al., 2010), we hypothesize that the g-dECM bioink can model the more aggressive characteristics of gastric cancer.

**FIGURE 3:**
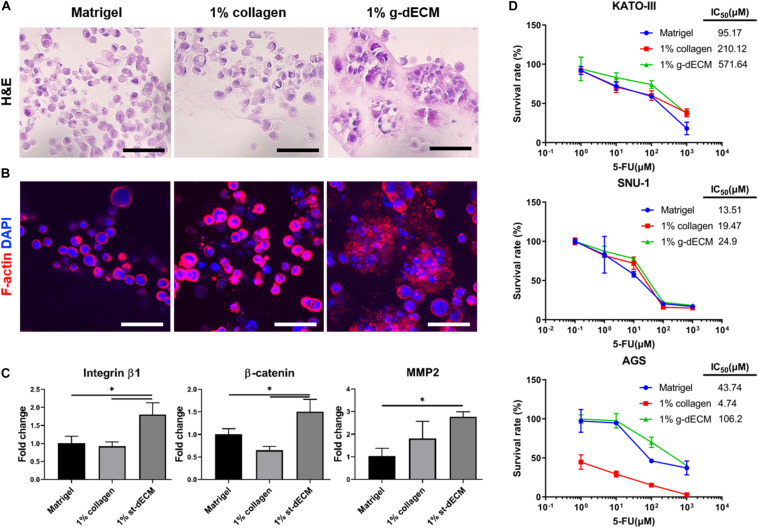
Evaluation of cell aggregation and aggressiveness of gastric cancer cells 3D printed in different bioinks. **(A,B)** Histology of gastric cancer cell-line (KATO-III) grown in Matrigel, 1% collagen, and 1% g-dECM on day 14. **(A)** H%E stained images **(B)** Immunofluorescence images. **(C)** Comparative gene expression analyses of integrin β1, β-catenin, and MMP2 on day 14. **(D)** Response of gastric cancer cell-line (KATO–III, SNU-1, and AGS) proliferation in Matrigel, 1% collagen, and 1% g-dECM bioink to 5-FU treatment. (*n* = 3) (scale, 50 μm, ^∗^*p* < 0.05).

To verify this hypothesis, we examined the presentation of fundamental gastric cancer cell behaviors in the g-dECM bioink, using Matrigel and collagen as representative controls. Here, Matrigel, composed of basement membrane components from tumor cell/tissues (Benton et al., 2014), was selected as it is the most widely used material for modeling the cancer environment. Conversely, Type I collagen—a biomaterial obtained from natural ECM components—was selected as the negative control for cancer behavior, as it shows high biocompatibility and is widely used for developing tissue models (Che et al., 2006; Yip and Cho, 2013). To ensure all experiments were conducted in identical conditions to Matrigel, which has a protein concentration of approximately 10 mg/ml, the concentrations of the g-dECM and collagen were set at 1 w/v%. Histological analysis was performed to study the morphological behavior of the KATO-III gastric cancer cell line, which is derived from gastric signet ring cell carcinoma (Takeuchi et al., 2012). Interestingly, although signet ring cells were observed in all three groups, both H&E staining (Figure 3A) and confocal imaging (Figure 3B) indicate that cells only aggregate in the 1% g-dECM bioink. No cell aggregates were observed in either Matrigel or collagen, suggesting that the g-dECM bioink is more effective in inducing cancer cell aggregation.

The expression of the tissue remodeling marker (MMP2), the cell–cell interaction marker (β-catenin), and the cell–ECM interaction marker (integrin β1), which are involved in gastric cancer cell aggregation and are used to characterize the aggressiveness of cancer cells, were also investigated. We observed that the expression of MMP2, β-catenin, and integrin β1 was significantly higher with the 1% g-dECM bioink than with the Matrigel and collagen (Figure 3C). These results indicate that gastric cancer cells showed more aggressive characteristics in the g-dECM than in the other biomaterials.

As cell adhesion molecules can play a crucial role in therapeutic resistance, we conducted drug tests by collating the response of cells in each biomaterial to 5-FU. Here, three different gastric cancer cell lines (SNU-1, KATO-III, and AGS) were encapsulated in each hydrogel, and cultured for 2 weeks. Then, 0–1,000 μM 5-FU was added for 2 days. As expected based on the increased expression of the marker genes, the IC_50_ values were higher in the 1% g-dECM group, with 6 and 2.7-fold increases noted for KATO-III, 1.8 and 1.3-fold increases noted for SNU-1, and 2.4 and 22.4-fold increases noted for AGS, in comparison to the values in Matrigel and collagen, respectively (Figure 3D). These figures indicate that culturing in the dECM bioink increased the drug resistance of gastric cancer cells. Thus, the proposed g-dECM bioink showed favorable feasibility for further applications in modeling gastric cancer.

### Regulating Cancer Behavior Using the Stiffness of the g-dECM Bioink

Cancerous growths are usually observed in stiffer tissue environments than the environments of normal tissues. Hence, it has been surmised that cancer cellular behavior is regulated based on ECM stiffness (Gkretsi and Stylianopoulos, 2018; Kalli and Stylianopoulos, 2018). In a previous *in vitro* study, to modulate the cancer cells, ECM stiffness was controlled by changing the protein density or the degree of hydrogel crosslinking. These controls subsequently activated cancer cell behavior, such as enhancing the integrin–ECM adhesion of plaque mechanosensors (Gauthier and Roca-Cusachs, 2018). Thus, we hypothesize that behavior of gastric cancer cells can be upregulated by increasing the concentration of the g-dECM bioink.

**FIGURE 4:**
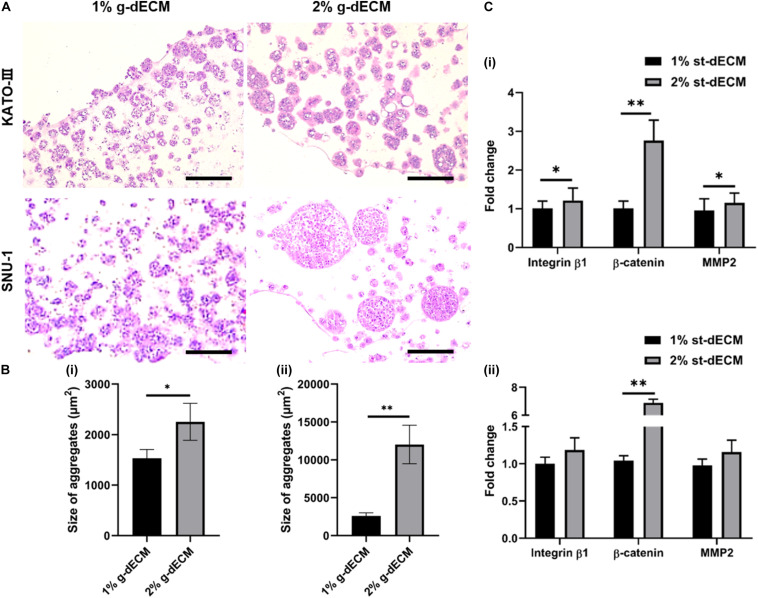
Extracellular matrix density regulates gastric cancer cell aggressiveness. **(A)** H&E stained image of gastric cancer cell-lines (KATO-III and SNU-1) grown in 1 and 2% g-dECM bioink on day 14. **(B)** Quantification of the size of cell aggregates. **(C)** Comparative analyses of gene expression on day 14 (scale, 200 μm; ^∗^*p* < 0.05, ^∗∗^*p* < 0.01).

To investigate this, we compared the behaviors of KATO-III and SNU-1 cells encapsulated in 1% g-dECM bioink with the behavior of the same gastric cell lines encapsulated in 2% g-dECM bioink. H&E staining showed that with both cell lines, the size of the cell aggregates were larger in the 2% g-dECM bioink than in the 1% g-dECM bioink (Figures 4A,B). From Figure 1Dii, under 1 radS^–1^, the storage modulus of the 1% g-dECM was 129.8 ± 54.7 Pa, whereas the storage modulus of the 2% g-dECM ink was 376.6 ± 156.1 Pa under 1 radS^–1^. This result thus indicates that the higher ECM stiffness caused the gastric cancer cells to form larger aggregates.

Further, we observed the expression of the cancer-related markers, MMP2, β-catenin, and integrin β1, which are associated with matrix stiffness and correlated with cancer cell invasion and metastasis (Karamichos et al., 2007; You et al., 2015). As expected, the levels of MMP2, β-catenin, and integrin β1 were upregulated with increased g-dECM bioink stiffness (Figure 4C); in the 2% g-dECM bioink, KATO-III showed significantly higher expressions of all three markers, whereas SNU-1 showed a significantly higher expression of β-catenin, and more modest increases in MMP2 and integrin β1 expression. Thus, the g-dECM matrix stiffness regulates remodeling gene expression, demonstrating that control of the aggression of gastric cancer cells is feasible.

### Effects of Cellulose Nanoparticles on Regulating the Mechanical Properties of g-dECM Bioink and Cellular Behavior

**FIGURE 5:**
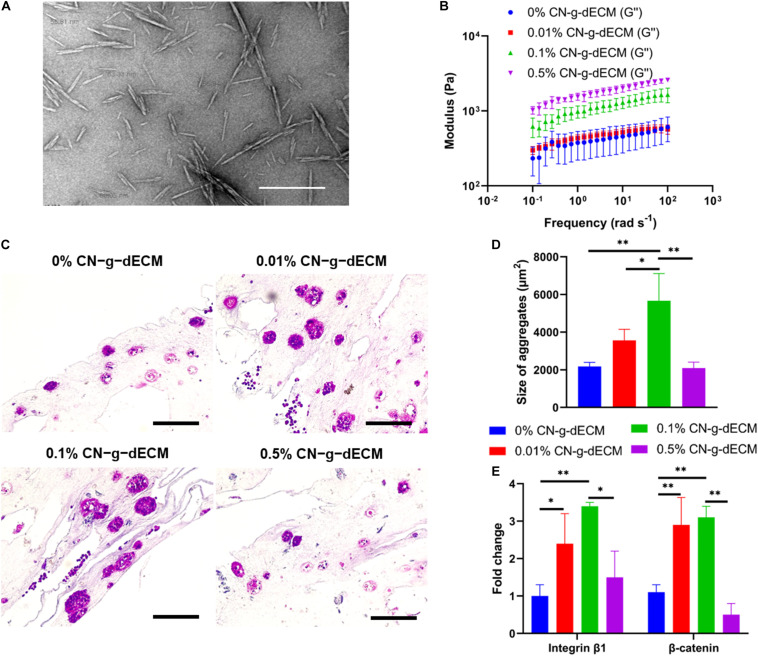
Enhancement of extracellular matrix density using cellulose nanoparticles regulates the aggressive behavior of gastric cancer cells. **(A)** TEM image of cellulose nanoparticles. **(B)** Dynamic modulus of CN-g-dECM bioink at 37°C. **(C)** H&E stained image of gastric cancer tumor grown in CN-g-dECM bioink on day 14. **(D)** Quantification of the size of cell aggregates. **(E)** Comparative analyses of gene expression on day 14 (scale, 200 μm; ^∗^*p* < 0.05, ^∗∗^*p* < 0.01).

In the previous section, it was demonstrated that increasing the density of g-dECM bioink stimulated more aggressive gastric cancer behavior. However, the 2% g-dECM bioink formulation is the maximum concentration achievable. Hence, to enhance its mechanical properties and provide a more biophysically reliable gastric cancer environment, we investigated the use of a cross-linker in addition to the bioink. Cellulose has been identified as a promising biopolymer with remarkable biological properties such as biocompatibility, biodegradability, and low toxicity (Luo et al., 2019). Therefore, in this study we used CN to further increase the mechanical strength of the g-dECM bioink. CN particles were prepared following the methods previously described in the literature (Kumar et al., 2017). The diameters of the prepared particles were observed to be in the 50–100 nm range using TEM (Figure 5A). The final concentration of the prepared CN solution was approximately 20%. The concentration of the CN-g-dECM bioink was set in the range 0–0.5 w/v%, i.e., we compared the behavior of g-dECM without CN, to the behavior of g-dECM mixed with CN up to a concentration of 0.5 w/v%.

The stiffness of the 2% g-dECM bioink improved with increases to the concentration of added CN (Figure 5B). To investigate the effect of the CN on cellular behavior, KATO–III was encapsulated in each bioink formulation. Primary thermal crosslinking was subsequently conducted by incubating at 37°C for 30 min, followed by secondary crosslinking, conducted by treatment with 100 × 10^–3^ m CaCl_2_. After culturing for 2 weeks, aggregated cells were observed in all groups through histological analysis (Figure 5C). The addition of the CN increased the size of aggregates from 2178.7 ± 210.7 μm^2^ in the 0% CN-g-dECM group, to 3563.8 ± 583.3 μm^2^, and 5666.5 ± 1440.1 μm^2^ in the 0.01% CN-g-dECM, and 0.1% CN-g-dECM groups, respectively. In contrast, the size of cell aggregates in the 0.5% CN-g-dECM group decreased to 2095.1 ± 313.0 μm^2^ (Figure 5D). These results indicate that adjusting the mechanical properties of the bioink using CN supplements can regulate cell aggregation. These observations were further corroborated using the expression of β-catenin and integrin β1, which are sensitive to stiffness (Samuel et al., 2011; Yeh et al., 2017). Up to a concentration of 0.1% CN in g-dECM bioink, where the largest aggregate sizes were observed, the levels of β-catenin and integrin β1 increased with an increase in the matrix stiffness. However, both gene expression and cell aggregate sizes were decreased in the 0.5% CN-g-dECM bioink (Figure 5E), suggesting an improper physical microenvironment for cell proliferation (Cavo et al., 2016). Thus, it can be surmised that an excessively high CN concentration results in an inordinately stiff cell environment that degrades cell properties.

These outcomes indicate that more aggressive cellular functions can be obtained by regulating the stiffness of the bioink using CN. Furthermore, an adequate biophysical environment for gastric cancer cells can be obtained by modulating the concentration of CN.

## Discussion

The behavior of gastric cancer cells is regulated by the surrounding environment (Ishimoto et al., 2014). Recognizing the importance of this variable, we developed a 3D cell printed gastric cancer model that uses a gastric specific bioink supplemented with cellulose nanoparticles to provide tissue-specific biochemical and biophysical stimulation of the environment for cancer cells. In our study, we observed that gastric cancer cells in the g-dECM bioink were highly aggregated, in contrast to those in collagen and Matrigel at the same concentration to consider the features of natural ECM. Further, we observed that marker genes related to cancer aggressiveness—MMP2, β-catenin, and integrin β1—were expressed at higher levels in the g-dECM bioink. These results indicated that the g-dECM bioink affects cellular functions, such as matrix remodeling, cell–ECM interaction, and cell–cell interaction, which lead to cancer progression (Kaushik et al., 2019). In addition, because drug resistance is an intrinsic behavior of cancer and plays an important role in developing cancer models (Gottesman, 2002), and organ microenvironment may affect the response to chemotherapy (Khanna and Hunter, 2005), we verified that the therapeutic resistance of gastric cancer cells was increased in the g-dECM bioink. Our findings demonstrate the efficacy of the g-dECM bioink as a drug testing material, in mimicking *in vivo* conditions that showed high drug resistance. These observations are attributed to the fact that tissue-specific bioinks can provide a tissue-specific environment for cancer cells (Tian et al., 2018) that promote cancer cell progression.

In addition, as already demonstrated in previous studies, native gastric cancer tissues are stiff, and this matrix stiffness regulates the behavior of encapsulated gastric cancer cells (Song et al., 2013; da Cunha et al., 2016). To reconstruct this *in vitro*, in this study, two methods were chosen to influence the surrounding biophysical environment to regulate the cellular function. In the first method, we increased the ratio of g-dECM to acetic acid in the g-dECM bioink, to enhance its mechanical properties. As well as the enlargement of cell aggregates, we observed that increasing the concentration of g-dECM in the bioink upregulated β-catenin and integrin β1. This implies that the proposed g-dECM bioink can provide a biochemically and biophysically appropriate microenvironment for culturing gastric cancer cells. In the second method, we used CNs, which have superior mechanical strength and excellent biocompatibility (Luo et al., 2019), to enhance the stiffness of the g-dECM bioink. An increase in the modulus of the bioink induced larger cell aggregates and higher expression of β-catenin and integrin β1, which indicates that ECM stiffness of the prepared structure regulates cell–cell interaction and cell–ECM interaction. Moreover, we observed that the 0.1% CN-g-dECM bioink provided the most suitable stiffness for the gastric cancer cells. This indicates that, with its more aggressive characteristic, 0.1% CN-g-dECM can be used to provide a more reliable clinically applicable predictor, compared to previous methods relying on 2D and 3D culture in Matrigel and collagen.

In addition, the g-dECM bioink can be used to fabricate arbitrary 3D structures using automated 3D cell-printing techniques that enable the deposition of various cell-laden bioinks at appropriate positions (Pati et al., 2014). Hence, using the developed bioink, 3D cell-printing techniques could enable the fabrication of more complex gastric cancer systems with different types of cells, such as fibroblasts and endothelial cells, that can provide an alternative to animal models. This is important, as it is increasingly clear that owing to cross-species differences, animal models do not accurately predict the human body’s response to drug testing. With their ability to mimic *in vivo* environments, and the automated model fabrication process, 3D cell-printed cancer models have become prominent candidates to replace animal models (Kang et al., 2020). Therefore, using our biochemically and biophysically improved bioink, we can fabricate more *in vivo* relevant gastric cancer systems in future studies.

## Conclusion

In this study, we developed a CN-g-dECM bioink for 3D cell printing a gastric cancer model. With respect to clinical study, the developed bioink has the advantages of providing a biochemically and biophysically appropriate microenvironment for analyzing gastric cancer cells. Compared to commercially available hydrogels such as Matrigel and collagen, gastric cancer cells in this bioink showed more aggressive characteristics, as confirmed by morphological, drug testing, and genetical analyses. Moreover, the inclusion of CN in the g-dECM bioink allows the regulation of the size of cell aggregates, and the expression of MMP2, β-catenin, and integrin β1, by controlling the stiffness of the cancer microenvironment. Further, cell-laden bioink can be patterned in the appropriate position using 3D cell printing techniques, meaning that it can be applied for fabricating complex gastric cancer systems.

## Data Availability Statement

The datasets presented in this study can be found in online repositories. The names of the repository/repositories and accession number(s) can be found in the article/Supplementary Material.

## Author Contributions

JK, JJ, and D-WC conceived the initial idea and designed the experiments. JK performed the experiments. JK and JJ analyzed the data and drafted the manuscript. JJ and D-WC guided the work and revised the manuscript and worked on funding acquisition. All authors have read and approved the final manuscript.

## Conflict of Interest

The authors declare that the research was conducted in the absence of any commercial or financial relationships that could be construed as a potential conflict of interest.
