# Supplementary material for: Controlling Cancer Cell Behavior by Improving the Stiffness of Gastric Tissue-Decellularized ECM Bioink With Cellulose Nanoparticles
Source: Front Bioeng Biotechnol. 2021 Mar 17;9:605819. doi: 10.3389/fbioe.2021.605819 (PMC8009980; doi:10.3389/fbioe.2021.605819)
Supplement: Supplementary file 1 [file Data_Sheet_1.docx]

Controlling cancer cell behavior by improving the stiffness of gastric tissue-decellularized ECM bioink with cellulose nanoparticles

Jisoo Kim^1^, Jinah Jang^1,2,3,4*^, Dong-Woo Cho^1,2,4*^

^1^School of Interdisciplinary Bioscience and Bioengineering, Pohang University of Science and Technology (POSTECH), Pohang, South Korea

^2^Department of Mechanical Engineering, Pohang University of Science and Technology (POSTECH), Pohang, South Korea

^3^Department of Creative IT Engineering, Pohang University of Science and Technology (POSTECH), Pohang, South Korea

^4^Institute of Convergence Science, Yonsei University, 50 Yonsei-ro, Seodaemun-gu, Seoul, 03722, Republic of Korea

*** Correspondence:**Prof. Jinah Jang
jinahjang@postech.ac.kr

Prof. Dong-Woo Cho
dwcho@postech.ac.kr

This supporting information includes supplementary data as below:

Figure S1. Image of Integrated Composite tissue/organ Building System (ICBS).


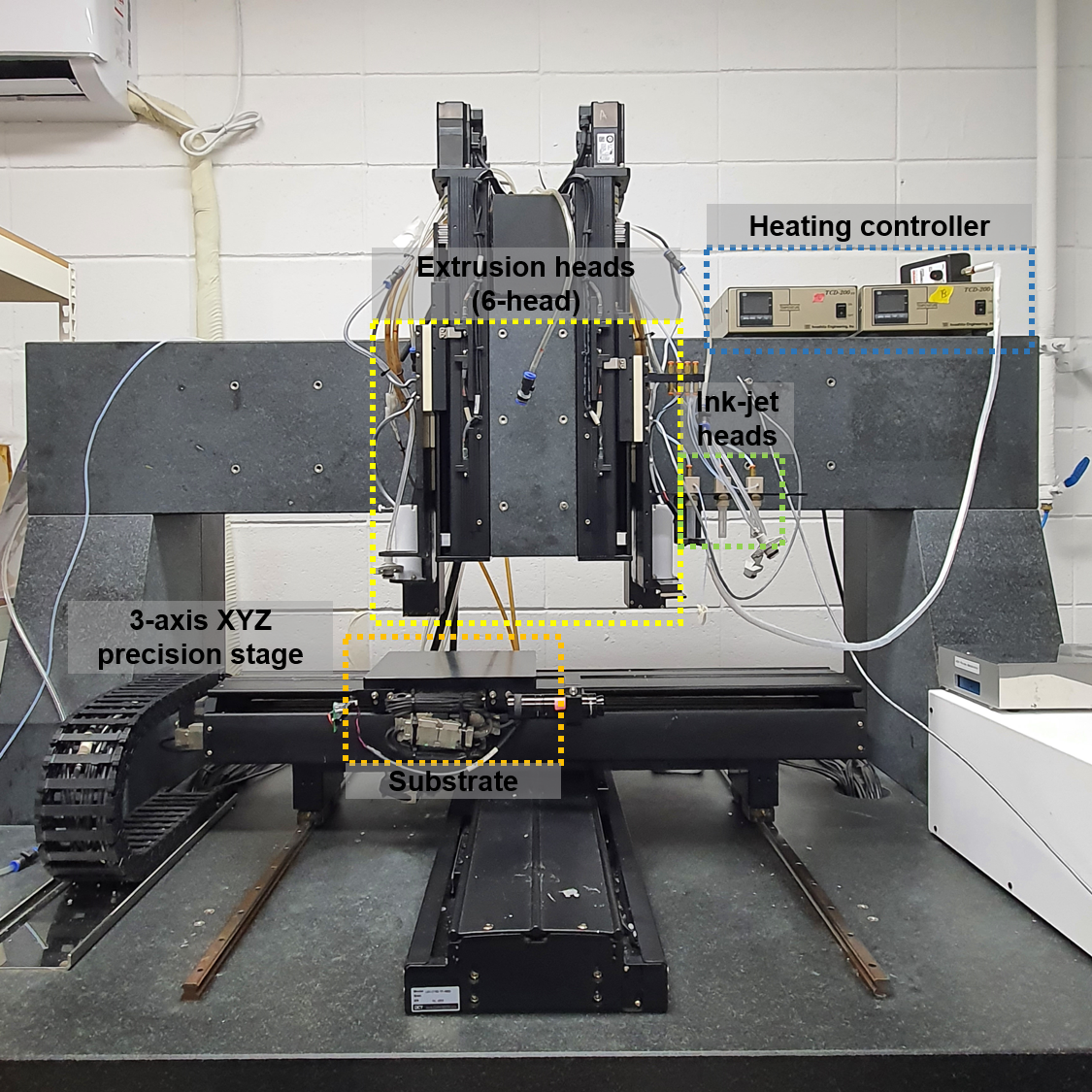


**Figure S1.** Image of Integrated Composite tissue/organ Building System (ICBS).
